# Hybrid Lipid-Polymer Bilayers: pH-Mediated Interactions between Hybrid Vesicles and Glass

**DOI:** 10.3390/polym12040745

**Published:** 2020-03-28

**Authors:** Keith L. Willes, Jasmyn R. Genchev, Walter F. Paxton

**Affiliations:** 1 Department of Chemistry and Biochemistry, Brigham Young University, Provo, UT 84602, USA; willes.keith@gmail.com; 2 Department of Chemistry and Biochemistry, Northern Arizona University, Flagstaff, AZ 86011, USA; jrg546@nau.edu

**Keywords:** polymersomes, hybrid vesicles, membrane bilayers, membrane properties, quartz crystal microbalance

## Abstract

One practical approach towards robust and stable biomimetic platforms is to generate hybrid bilayers that incorporate both lipids and block co-polymer amphiphiles. The currently limited number of reports on the interaction of glass surfaces with hybrid lipid and polymer vesicles—DOPC mixed with amphiphilic poly(ethylene oxide-b-butadiene) (PEO-PBd)—describe substantially different conclusions under very similar conditions (i.e., same pH). In this study, we varied vesicle composition and solution pH in order to generate a broader picture of spontaneous hybrid lipid/polymer vesicle interactions with rigid supports. Using quartz crystal microbalance with dissipation (QCM-D), we followed the interaction of hybrid lipid-polymer vesicles with borosilicate glass as a function of pH. We found pH-dependent adsorption/fusion of hybrid vesicles that accounts for some of the contradictory results observed in previous studies. Our results show that the formation of hybrid lipid-polymer bilayers is highly pH dependent and indicate that the interaction between glass surfaces and hybrid DOPC/PEO-PBd can be tuned with pH.

## 1. Introduction

The formation of supported bilayers over rigid materials is an essential step towards producing a wide range of biomimetic surfaces and devices including membrane-based biosensors [1,2,3]. Sensors that incorporate biomacromolecules involved in receptor binding or molecular traffic across membranes would be highly tunable and have the potential for sensing with the sensitivity and specificity of biological structures. Indeed, biosensors based on lipid bilayer membranes have demonstrated ways to interrogate membrane-based biochemical processes optically [4], electrically [2,3,5], and acoustically [6,7,8,9].

A critical aspect in the design of bilayer-based biosensors is the formation of robust and well defined lipid bilayers over rigid supports. In the years since first described by Tamm et al. [10], the formation of lipid bilayers on siliceous surfaces is a thoroughly studied phenomenon and the literature presents a great number of insights into the process. For example, the interaction between lipid vesicles and glass substrates is highly dependent on pH due to the changes it induces on the surface charge of the substrate [11], resulting in outcomes that range from adsorbed vesicles to supported lipid bilayers (SLBs). In addition to pH, other factors that affect vesicle-substrate interaction include temperature, cation valency, osmotic pressure, vesicle concentration and flow conditions [12,13,14,15].

While lipid bilayers are amenable to the reconstitution of membrane proteins, such bilayers suffer from mechanical and shear instabilities that make them ill-suited for practical device applications [16,17]. A number of strategies for addressing these instabilities have been demonstrated, including polymerization [18,19,20] or conjugation [14,21] of PEG to the lipids that form the bilayer membranes. Generating supported polymer bilayers and supported hybrid lipid/polymer bilayers (SHBs) offers another practical approach towards robust and stable biomimetic platforms. Indeed, there is a growing interest in understanding and exploiting the formation of SHB films in the past few years [22,23,24,25,26,27], including the use of amphiphilic polymers to complement the library of conventional lipids. Such hybrid materials have recently been highlighted as an important development toward biomimetic interfaces [28,29]. Hybrid membrane materials would enable tunable membrane permeability and diffusivity [30], and greater hydrodynamic stability (e.g., vesicles that do not aggregate over time) [26]. In addition, hybrid lipid/polymer membranes are amenable to the reconstitution of membrane proteins [31,32], and may even enhance their stability [33], including stability in solid supported membranes [23]. These properties are all, in principle, tunable by adjusting the composition of the hybrid vesicles, and may lead to more versatile platforms for robust biosensors that operate by mimicking the properties and functions of biological membranes.

The limited number of reports on the interaction of hybrid and polymer vesicles with glass surfaces describe substantially different conclusions under very similar conditions (i.e., same pH)—from bilayer formation, to irreversible vesicle adsorption, to no interaction at all [22,23,26]. The range of reported results is possibly due to insufficient vesicle substrate interactions which—as demonstrated for lipid-only systems [11]—can be mediated by varying buffer pH. To our knowledge there has been no systematic study of the effects of buffer pH on hybrid vesicle adsorption and fusion. Understanding these effects is critical to preparing biomimetic interfaces that incorporate stability-enhancing polymer components. 

In this study, we varied vesicle composition and buffer pH in order to generate a broader picture of spontaneous hybrid lipid/polymer vesicle interactions with rigid supports. Laying down a basic understanding of these new hybrid bilayers will provide valuable insight for future researchers in this area. In this paper, we demonstrate that the formation of supported films from hybrid lipid/polymer materials over glass surfaces is highly pH dependent. Vesicles composed of varying fractions of poly(ethylene oxide–*b*–butadiene) (EO_22_Bd_33_) and the lipid 1,2-dioleoyl-sn-glycero-3-phosphocholine (DOPC) form films consistent with (i) bilayers, (ii) adsorbed vesicles, and (iii) a mixture of the two depending on the composition of the vesicles and the pH of the supporting buffer solution. These results highlight the important role of pH in the formation of polymer and hybrid lipid-polymer bilayers on solid supports, particularly under weakly acidic or neutral conditions. 

## 2. Materials and Methods

To investigate the interaction between hybrid vesicles and glass surfaces, we measured the size and the effective zeta potential, ζ, of the vesicles and glass surfaces under our experimental conditions. Then, we monitored the interaction between vesicles and surfaces using quartz crystal microbalance with dissipation under different pH conditions.

### 2.1. Materials

Polymer and lipid reagents were purchased from commercial venders and stored and used according to the recommendations of the vendor: 1,2-dioleoyl-sn-glycero-3-phosphocholine (DOPC; Avanti Polar Lipids); poly(ethylene oxide-b-butadiene) (EO_22_Bd_33_; P40494B, Polymer Source). Deionized (DI) water was prepared using a Millipore Synergy UV-R water purification system. Tris buffer solutions (10 mM tris(hydroxymethyl)aminomethane and 100 mM NaCl) were prepared and titrated to their appropriate pH using HCl or NaOH, while controlling for osmolality (200 +/−10 mOsm). Silica microspheres (0.163 micrometers, Bangs Laboratories) were diluted to 1 mg mL^−1^ in Tris buffer solutions at pH values ranging from 2 to 12.

### 2.2. Preparation of Hybrid Vesicles

DOPC and EO_22_Bd_33_ were dissolved in chloroform (1 mg mL^−1^) at polymer molar ratios of 0%, 10%, 25%, 50%, 75%, 100%. The solvent was evaporated off using a Buchi R-300 rotovap at 350 mbar pressure, 120 RPM and a 40 °C water bath. The evaporated samples were stored under vacuum for at least one hour and rehydrated to 1 mg mL^−1^ in Tris buffer solution (pH = 7). The rehydration process was aided by heating in a hot water bath (40 °C) and vortexing vigorously. Unilamellar vesicles were then formed by extrusion using an Avanti extruder with a 50 nm polycarbonate membrane (Avanti). After extrusion, vesicles were diluted to 0.1 mg mL^−1^ using Tris buffer solution, at increments of 1 pH unit from a pH value of 2 to 12.

### 2.3. Zeta Potential from Dynamic Light Scattering

A Malvern Nano ZS (ZEN 3600) was used to acquire size distribution and zeta potential measurements of the extruded vesicles. For DLS measurements the extruded vesicles were diluted to 0.1 mg mL^−1^ in Tris buffer pH 7 and pipetted into a disposable cuvette. For zeta potential measurements the extruded vesicles were diluted in Tris buffer solutions of pH = 2–12 and injected into a disposable capillary cell cuvette. In order to gauge the effect of pH on the charge of glass, silica microspheres (1.0 mg mL^−1^) in Tris buffer solutions of pH = 2–12 were similarly prepared and analyzed.

### 2.4. QCM-D Measurements

A Q-sense E4 instrument (Nanoscience Instruments, Phoenix, AZ, USA) was used to monitor the adsorption behavior of hybrid vesicles onto borosilicate coated AT-cut quartz crystals (QSX336, Nanoscience Instruments). Before use in any experiments, the sensors were thoroughly rinsed with the following solution in order, DI water, 2% Hellmanex^®^ III, DI water, isopropyl alcohol, DI water. They were then dried under a vigorous stream of nitrogen gas and then cleaned with RF/air plasma for 5 min immediately prior to use. The prepared vesicles (0.1 mg mL^−1^) were introduced into the flow module using a peristaltic pump set at 200 µL/min. For all hybrid vesicle compositions, frequency and dissipation changes were monitored in solutions with pH levels ranging from 2 to 12 in increments of 1 pH unit. All odd overtones were monitored from 1 to 13. Prior to collecting QCM-D data, the borosilicate sensor was equilibrated with the Tris buffer solution of the desired pH for at least 15 min before introducing the vesicle suspension of the same pH. This use of identical solutions for equilibration and experiment minimized slight fluctuations in QCM-D response due to solutions of different compositions and ensured that the changes we observed were due to the interaction between vesicles in suspension and the sensor crystal. Stable equilibrium of Δ*f/n* (where *n* is the overtone number) and Δ*D* were taken as the point at which the Δ*f/n* changed less than 1 Hz over 1 min, though both continued to change at a much smaller rate in some experiments. Film thicknesses were estimated using the Sauerbrey equation [34] or frequency and dissipation data using a frequency dependent viscoelastic model (Broadfit, Dfind software, Nanoscience Instruments).

## 3. Results and Discussion

### 3.1. Vesicle Preparation and Characterization

**Figure 1:**
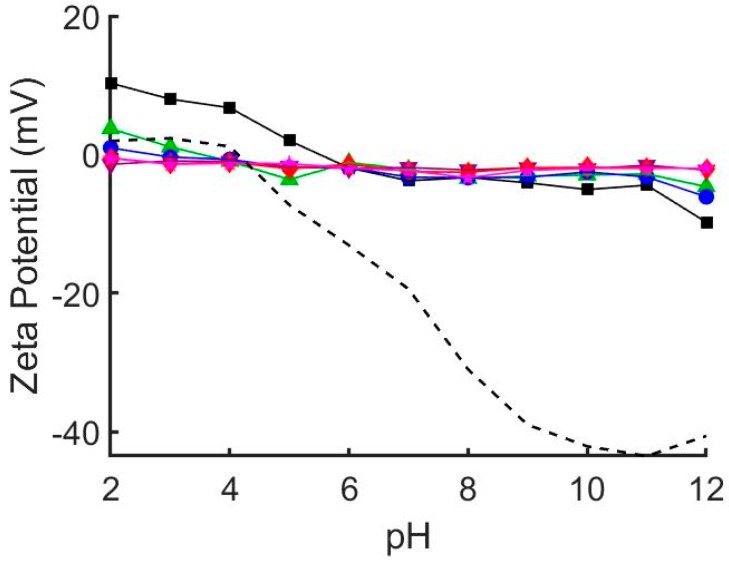
Zeta potential of glass beads (dashed line) and hybrid 1,2-dioleoyl-sn-glycero-3-phosphocholine (DOPC)/EO_22_Bd_33_ vesicles at varying pH. 0 mol % polymer (black squares), 10 mol % polymer (green triangles), 25 mol % polymer (blue circles), 50 mol % polymer (purple inverted triangles), 75 mol % polymer (red diamonds), 100 mol % polymer (pink stars). The zeta potential of pure DOPC vesicles depends greatly on solution pH. This pH dependence is greatly diminished by addition of EO_22_Bd_33_. At and above 50 mol % EO_22_Bd_33_ vesicle zeta potential is unaffected by solution pH. See also Figures S1 and S2.

We prepared a range of hybrid DOPC/EO_22_Bd_33_ suspensions with increasing amounts of EO_22_Bd_33_—0, 10, 25, 50, 75, and 100 mol % polymer—and characterized them by dynamic light scattering (DLS) and zeta potential analysis (Table 1). Notably, the size of the vesicles after extrusion increased with increasing polymer fraction, and 100 mol % polymer vesicles were roughly double the size of the lipid-only vesicles. The zeta potential, ζ, of suspended vesicles in each sample was also measured at pH = 7 and over a range of pH values (Figure 1, see also Figures S1 and S2 in supplementary materials) corresponding to the pH of suspensions used in the QCM-D adsorption experiments (see below). The ζ of DOPC vesicles was sensitive to changes in pH, varying from +10 to −10 mV across a pH range of 2 to 12. This sensitivity decreased as the mole fraction of polymer increased, and ζ of 100% polymer vesicles over the same pH range only varied from approximately 0 to −2 mV.

To estimate the electrostatic interaction between the vesicles and the glass substrate, we measured the pH-dependent ζ of SiO_2_ glass beads under conditions identical to those used for QCM-D experiments (i.e., same buffer concentration and pH). Our results (Figure 1, see also Figures S1 and S2) mirror those published previously indicating that the zeta potential of SiO_2_ glass [35] and borosilicate glass [36] is highly pH dependent. Notably, the point of zero charge was shifted to pH = 4 (compared to ~2 in unbuffered solution) and ranged from slightly positive at low pH (+2 mV at pH = 2) to significantly negative at high pH (−43 mV at pH = 11).

Our vesicle characterization results indicate that polymer fraction has significant effects on the properties of hybrid vesicles. The linear relationship (R^2^ = 0.980) between hydrodynamic radius and polymer fraction suggests increased mechanical stability of vesicles containing polymer. As the polymer fraction is increased, vesicles become more resistant to being rearranged into smaller structures when extruded. The effect is rather significant, resulting in an 85% increase in size between pure EO_22_Bd_33_ and DOPC vesicles, which correlates to a 6-fold increase in volume. The resistance to re-sizing by extrusion is likely due to the enhanced cohesive energy density keeping polymer chains together in polymersomes, which can be 2–20× greater than liquid-phase lipid vesicles [37,38]. There were also considerable changes in the zeta potential of hybrid vesicles. The zeta potential of DOPC vesicles is sensitive to changes in pH, and as polymer fraction increased this sensitivity diminished until at 50% polymer, there was virtually no response to changes in pH. This suggests that PEO covers the external surface of hybrid vesicles and shields the proton-exchangeable lipid head-groups subject to changes in pH.

### 3.2. pH Dependence of Lipid Vesicle–Borosilicate Interaction

**Figure 2:**
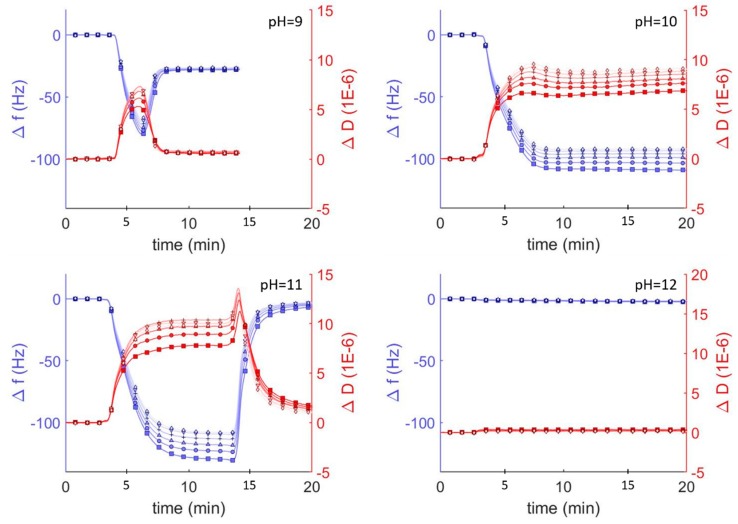
QCM-D monitoring of overtone normalized frequency change (Δ*f/n*; **blue** traces and axes) and dissipation changes (Δ*D*; shown in **red**) for lipid vesicles interacting with plasma cleaned borosilicate glass. After establishing a baseline buffer solution measurement, vesicles composed of DOPC (0.1 mg mL^−1^) in Tris buffer at varying pH were injected into the QCM module. Showing the 3rd through 13th overtones, 3rd (□), 5th (○), 7th (∆), 9th (+), 11th (inverted triangles), 13th (◊).

To investigate the interaction between hybrid vesicles and glass surfaces, we monitored the interaction between vesicles and surfaces using quartz crystal microbalance with dissipation (QCM-D) under different pH conditions. In the area of vesicle adsorption behavior, QCM-D is a well established technique which monitors the changes in resonant frequencies (Δ*f*) and energy dissipation (Δ*D*), which correspond to changes in mass and viscoelasticity, respectively, of adsorbed films on a harmonic oscillator in real time [15]. These changes can be related to the thickness and the viscoelastic and hydrodynamic properties of the adsorbed layers. We exposed borosilicate-coated QCM-D sensors to a suspension of DOPC vesicles in Tris buffer solution adjusted to different pH conditions and monitored the frequency and dissipation at each of the odd overtones (Figure 2). At pH<9, we observed a decrease followed by an increase in overtone-normalized frequency, Δ*f/n* (where *n* is the overtone number), to a stable equilibrium of −26 Hz after 5 min. These changes in frequency corresponded to a dramatic increase and then a decrease in acoustic dissipation to an equilibrium value of Δ*D* = 1 × 10^−6^, indicating a rigid film consistent with the adsorption-then-fusion of lipid vesicles to form an SLB. Performing the same experiment with vesicles in solutions at higher pH revealed three additional previously reported [11,15] pH-mediated QCM-D behaviors consistent with irreversible vesicle adsorption (pH = 10), reversible vesicle adsorption (pH = 11), and no adsorption (pH = 12).

SLBs can be easily distinguished from adsorbed lipid vesicles by comparing the Δ*f/n*, Δ*D*, and estimated film thicknesses. The final frequency and dissipation of pure DOPC bilayers on borosilicate glass is fairly constant between pH = 2 and 9 (approx. −26 Hz and 1 × 10^−6^, respectively). The average thickness of these adsorbed lipid bilayer films calculated using the Sauerbrey equation [34] (valid for rigid materials) was ~5.4 nm, consistent with lipid bilayer films. As the pH increased above 9, the vesicle-substrate interaction was not sufficient to induce vesicle fusion and the adsorbed lipid films became more viscoelastic, indicating the adsorption of lipid vesicles. Because the Sauerbrey relationship is only valid for rigid (not viscoelastic) films where ΔD < 1 × 10^−6^, we also estimated film thicknesses by fitting the frequency and dissipation data from different sensor crystal harmonics to a viscoelastic model (see Materials and Methods). Using this model, we determined the average thickness of a lipid bilayer at pH = 7 was approx. 6.6 nm, compared to ~5.4 nm using the Sauerbrey model. At higher pH values, the average thickness was 23 nm at pH = 10, where *irreversible* vesicle adsorption occurs, and 26 nm at pH = 11, where reversible vesicle adsorption occurs.

DOPC vesicles interact differently on silica surfaces depending on pH, resulting in the formation of supported bilayers, adsorption of vesicles, or the complete lack of interaction. DOPC bilayer formation under neutral pH follows a well known^13^ two-step process of vesicle adsorption and fusion to form uniform bilayers. In the first step vesicles adsorb to the surface and in the second step vesicle-vesicle and vesicle-substrate interactions combine to induce fusion and rupture to form SLBs. The vesicle fusion step requires a critical surface coverage of vesicles and can be observed by QCM characterized as an initial steep drop in frequency—indicating adsorbed vesicles at Δ*f/n* < −26 Hz—followed by an increase in frequency—indicating the formation of the bilayer and a Δ*f/n* of approx. −26 Hz. This initial drop in frequency is much less obvious at pH = 2 (−30 Hz) than at pH = 9 (−80 Hz), and indicates that vesicle fusion at low pH occurs at a lower surface coverage. This behavior has been attributed to a decrease in electrostatic repulsions between the glass and the vesicles as the pH is lowered, allowing attractive van der Waals interactions to drive bilayer formation [11]. Our analysis of zeta potential as a function of pH, which shows significantly attenuated negative potentials at low pH (Figure 1) supports this conclusion. Furthermore, it explains the complete lack of vesicle interaction at pH > 9, where both the vesicles and the glass have significant negative zeta potential that repel each other and prevent even vesicle adsorption under basic conditions.

### 3.3. Behavior of Lipid, Polymer, and Hybrid Vesicles at Neutral pH

**Figure 3:**
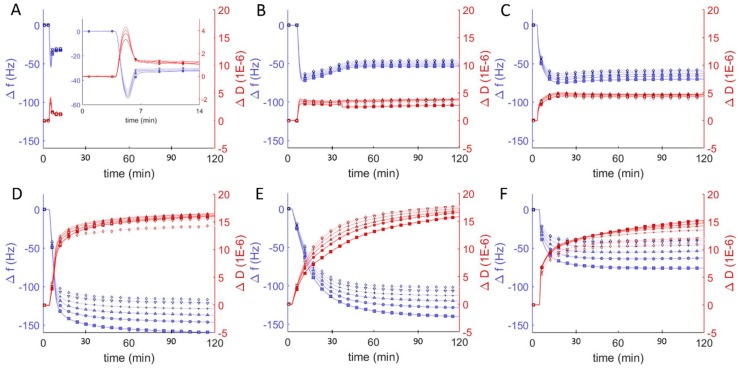
Frequency change (Δ*f/n*, blue) and dissipation (Δ*D*, red) measurements from QCM-D monitoring of hybrid polymer/lipid vesicles in Tris buffer (pH = 7) interacting with plasma cleaned borosilicate glass, showing data from the 3rd through 13th harmonics, 3rd (□), 5th (○), 7th (∆), 9th (+), 11th (inverted triangles), 13th (◊). After establishing a baseline buffer solution measurement, vesicles composed of 100 mol % DOPC (**A**) and EO_22_Bd_33_ in varying amounts: 10 (**B**), 25 (**C**), 50 (**D**), 75 (**E**), 100 (**F**) mol % were introduced into the sample QCM-D sample chamber.

Similar studies were performed at pH = 7 to probe the interaction between vesicles composed of DOPC and the amphiphilic block copolymer of poly(ethylene oxide-b-butadiene) (EO_22_Bd_33_). Hybrid vesicles with a polymer fraction of 10 mol% exhibited changes in frequency and dissipation similar to those of the pure DOPC vesicles: a decrease followed by an increase in resonant frequency, and corresponding changes in dissipation (Figure 3B). In contrast to the DOPC vesicles, the stable equilibrium changes in frequency and dissipation were larger for the hybrid vesicle, −50 Hz and 4 × 10^−6^ respectively, and this equilibrium was achieved after ~45 min, a much longer period compared to the equilibration time between lipid vesicles interacting with borosilicate sensors. Furthermore, with the increase in energy dissipation, the changes in frequency for the different overtones began to spread out, indicating an increased viscoelasticity of the adsorbed film [39]. The changes in Δ*f/n* and Δ*D* for the 25 mol % vesicles were similar (Figure 3C), but slightly greater and took even longer to stabilize (60 min). 

As the molar ratio of polymer to lipid in the vesicles increased to 50%, we observed large changes in both frequency and dissipation (magnitude of Δ*f/n* > 150 Hz and Δ*D* > 14 × 10^−6^), suggesting a dramatic change in the morphology of the adsorbed material that is not consistent with the formation of a uniform bilayer. Above 50 mol % polymer, the magnitude of the frequency changes decreased as the polymer fraction increased even while the dissipation remained essentially unchanged, indicating a decreasing adsorption of high-polymer content vesicles to the surface. 

Differences in frequency, dissipation, and film thickness provided important criteria for distinguishing between SLBs and adsorbed vesicles at different pH. In particular, SLBs are characterized by a dissipation < 1 × 10^−6^ and can be easily distinguished from adsorbed lipid vesicles, with dissipation > 5 × 10^−6^. Hybrid lipid-polymer bilayers on the other hand are expected to behave viscoelastically, due in part to the longer and more disordered and viscoelastic PBd chains, and the extended PEO chains, which can couple with surrounding water. This increased viscoelasticity is expected to manifest itself as an increased dissipation relative to SLBs. We previously observed SHBs on glass by atomic force microscopy and correlated their formation to a substantial increase in dissipation by QCM-D: 4–7 × 10^−6^ [22]. Reimhult et al. also observed a similar increase for bilayers formed from hybrid vesicles containing a shorter PEO-PBd polymer (2.2 kD compared to our 2.8 kD) [23]. Our results are consistent with these previous reports and we concluded that 10 and 25 mol % polymer vesicles formed supported bilayers with dissipation changes on the order of 5 × 10^−6^ at neutral pH. The QCM-D data for the interaction of borosilicate with hybrid vesicles that had higher polymer compositions (50, 75, 100 mol %) were more consistent with the dissipation for a heterogeneous film of adsorbed vesicles (ΔD > 10 × 10^−6^), which we observed previously as well [22]. For 100 mol %, the adsorption was substantially less than for 50 and 75 mol %, but with comparable dissipation. This result is strongly suggestive of adsorbed vesicles that couple hydrodynamically to the surrounding solution (i.e., exhibit more drag) [40]. 

### 3.4. Behavior of Lipid, Polymer, and Hybrid Vesicles over a pH Range

**Figure 4:**
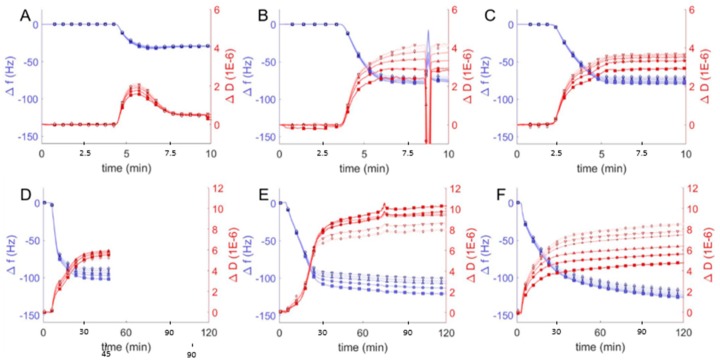
Frequency change (Δ*f/n*, blue) and dissipation (Δ*D*, red) measurements from QCM-D monitoring of hybrid polymer/lipid vesicles in Tris buffer (pH = 2) interacting with plasma cleaned borosilicate glass, showing data from the 3rd through 13th harmonics, 3rd (□), 5th (○), 7th (∆), 9th (+), 11th (inverted triangles), 13th (◊). After establishing a baseline buffer solution measurement, vesicles composed of 100 mol % DOPC (**A**) and EO_22_Bd_33_ in varying amounts: 10 (**B**), 25 (**C**), 50 (**D**), 75 (**E**), 100 (**F**) mol % were introduced into the sample QCM-D sample chamber. The spurious peaks in Δ*f/n* and Δ*D* at ~8 min in (B) are due to an air bubble which passed through the QCM-D chamber.

One puzzle from our previously reported experiments [22] was the difficulty in adsorbing 100% polymer vesicles on glass surfaces. Polymer vesicle adsorption and fusion at pH = 7 was not possible on glass surfaces prepared by RF/air plasma treatment, but polymer vesicles irreversibly adsorbed onto those same surfaces if they were first cleaned with an oxidizing strong acid solution (HCl/H_2_O_2_). Similarly puzzling, it has been reported that polyethylene glycol both adsorbs irreversibly [41] and also does not adsorb at all [42] on clean silica in neutral (pH ≈ 7) buffer solutions. The natural solution to this puzzle was to explore the adsorption behavior of polymer and hybrid vesicles as a function of pH. Using vesicles with a range in composition from pure DOPC to pure EO_22_Bd_33_ (10, 25, 50, 75, and 100 mol % polymer) we probed the interaction of hybrid vesicles with glass under varying pH conditions (2–12) by measuring changes in frequency and dissipation (Figure 4).

We first compared QCM-D data from hybrid vesicles interacting with glass at pH = 2 to the results obtained at pH = 7 (see above). The significant changes in frequency indicated substantial adsorption of hybrid and polymer vesicles at pH = 2 (Figure 4) compared to pH = 7 (Figure 3), but it was not immediately obvious whether the vesicles fused to create supported bilayers or remained as a heterogeneous film of adsorbed vesicles. To distinguish between different adsorption morphologies, we compared the changes in frequency and dissipation and the film thicknesses. The large frequency changes observed for favorable vesicle interactions with glass correspond to changes in dissipation as well and together can be used to deduce the morphology of the resulting films, either SHBs or adsorbed vesicles. As discussed above, SLBs are characterized by Δ*f/n* ≈ −26 Hz and Δ*D* < 1 × 10^−6^, while heterogeneous films of adsorbed vesicles have Δ*f/n* and Δ*D* significantly higher. We observed similar behavior in QCM-D response for hybrid and polymer vesicles at pH = 2 compared to pH = 7. For 10 and 25 mol % hybrid vesicle interactions were qualitatively similar. The increase in mass without a corresponding change in dissipation suggests more densely packed bilayers with comparable viscoelastic properties at lower pH, though we cannot rule out the possibility of heterogeneous films consisting of both bilayers and adsorbed vesicles.

For vesicles with polymer fractions of 50 and 75 mol %, we noted changes in frequency that corresponded to significant decreases in dissipation. These changes echoed similar changes in frequency and dissipation indicative of the transition from lipid vesicle adsorption at high pH to SLB formation at lower pH (Figure 2). Our interpretation, therefore, is that hybrid vesicles formed more viscoeslastic films of adsorbed hybrid vesicles at pH = 7 and more rigid supported bilayers at pH = 2 (though still viscoelastic relative to SLBs).

The difference in the behavior of polymer vesicles at pH = 7 and pH = 2 was striking: the QCM-D response of polymer vesicles interacting with glass at pH = 7 is marginal, but at pH = 2, substantial adsorption of vesicles was observed, as indicated by a much larger frequency change. The large changes in the dissipation due to polymer vesicle interaction at different solution pH (i.e., Δ*D* < 9 × 10^−6^ at pH = 2 vs. Δ*D* ~15 × 10^−6^ at pH = 7) reflects a more compact polymer film, in spite of greater changes in frequency, which correspond to increased adsorbed mass. We interpret these changes in QCM-D response as indicating homogenous bilayers, or possibly a mixture of polymer bilayers and adsorbed vesicles.

**Figure 5:**
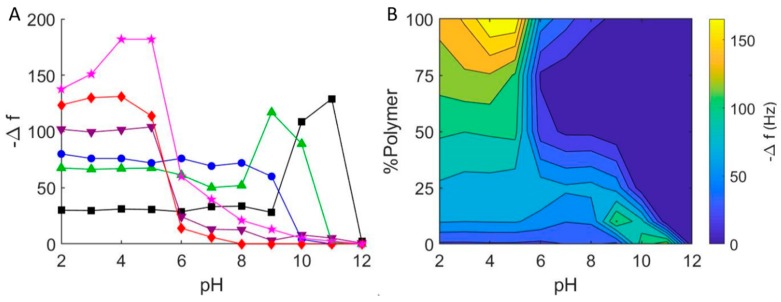
(**A**) Final frequency changes for the interaction of hybrid DOPC/EO_22_Bd_33_ vesicles with borosilicate glass at varying pH. 0 mol % polymer (black squares), 10 mol % polymer (green triangles), 25 mol % polymer (blue circles), 50 mol % polymer (purple inverted triangles), 75 mol % polymer (red diamonds), 100 mol % polymer (pink stars). (**B**) Graphical representation of QCM-D measurements of hybrid DOPC/EO_22_Bd_33_ vesicles at varying pH. Data points were taken after a stable frequency was reached.

In addition to using QCM-D to study vesicle-glass interactions at pH = 7 and pH = 2, we performed similar experiments over a pH range of 2–12, which included repeating the pH = 7 series, and compared the final stable equilibrium changes in frequency and dissipation as a function of pH and vesicle composition (Figure 5A). As with lipid vesicles, pH plays a significant role in the interaction between lipid-polymer hybrid vesicles and borosilicate glass. For pure DOPC vesicles, as mentioned above, our results echoed those of Cho et al.:^11^ changes in resonant frequency at pH = 2–9 are consistent with bilayer formation via vesicle adsorption and subsequent fusion (Δ*f/n* ≈ −26 Hz). Increasing pH further, we observed a greater decrease in frequency and increase in dissipation consistent with irreversible vesicle adsorption (pH = 10), reversible vesicle adsorption (pH = 11), and no adsorption (pH > 12). We observed similar pH-dependent results for lipid-polymer hybrid vesicles, with some important differences and consistent trends. Notably, as the EO_22_Bd_33_ fraction in the hybrid vesicles was increased, the transition between adsorption and no adsorption shifted towards lower pH. That is, there was no significant adsorption at pH > 12 for DOPC vesicles, but this critical pH above which adsorption was not observed (pH of no-adsorption) shifted to pH = 11 for 10 mol %; and pH = 10 for 25 mol % polymer vesicles. 

At a lower pH, we saw different results that depended on the composition of the vesicles. Below the critical adsorption pH for 10 mol % polymer (pH = 11), we observed frequency and dissipation changes that were consistent with adsorbed vesicles at pH = 9–10. Below pH = 9, we noted QCM-D response very similar to that obtained for SHBs (see above). For 25 mol % hybrid vesicles, the results were very similar, except that there was no discernable region of adsorbed vesicles: below pH = 10, we only observed changes in Δ*f/n* and Δ*D* consistent with supported bilayers. Hybrid vesicles composed of 50 and 75 mol % polymer showed very little interaction at neutral pH. This was in contrast to our first trial (Figure 3), where we noted significant vesicle-glass interactions for 50 mol % and 75 mol % hybrid vesicles. Nevertheless, the adsorption of hybrid vesicles with higher polymer fractions was strikingly more significant below pH = 7 than above, and this was evident for 100 mol % polymer vesicles as well. 

The substantial interaction (i.e., greater magnitude change in frequency) between polymer hybrid vesicles with higher polymer fractions (50, 75 and 100 mol %) and glass near pH = 7 demonstrated the puzzling inconsistency in interaction between polymer vesicles and glass—sometimes adsorption and sometimes none at all. As we changed the pH, a clearer picture of its effect on hybrid vesicle interactions with glass emerged: increasing the pH resulted in even smaller Δ*f/n*, indicating even less vesicle-glass interaction, while decreasing it resulted in significant adsorption (Δ*f/n* < −100 Hz) of high-polymer content hybrid vesicles below pH = 5. This greater interaction is due to the higher probability of hydrogen bond formation under more acidic conditions between (i) the hydrogen bond acceptors in the PEO corona of the polymer-containing vesicles and (ii) the hydrogen bond donating silanols (i.e., the protonated silicates) of glass [43]. Taken together, the pH dependence of PEO-containing hybrid vesicles is clear: significant adsorption is possible at low pH, but there is virtually no adsorption above pH = 8.

With the results of our experiments, we prepared a contour plot that interpolates the QCM data to give a qualitative representation of the interaction between hybrid lipid-polymer vesicles and glass (Figure 5B). This plot conveys the parameter space where we expect to see hybrid vesicle interactions on glass surfaces. In particular, it is evident that it is not likely possible to form supported bilayers from vesicles that have a high fraction of PEO at high pH, and in some cases, even at neutral pH. In addition, the 100 mol % polymer vesicles appear to form films of adsorbed vesicles at pH = 4, and bilayers of PEO-PBd possible only at lower pH.

To complement our analysis of QCM-D response to pH-dependent hybrid vesicle adsorption on glass, we estimated film thicknesses of hybrid lipid-polymer films formed under different pH conditions (Table 2). For lipid films at pH = 2, the Sauerbrey model for rigid homogenous films yielded estimated bilayer film thicknesses of 5.3 nm, comparable to estimates for bilayer films at pH = 7 (i.e., 5.4 nm). PEO-PBd in hybrid vesicles significantly increased Sauerbrey estimated thicknesses of supported bilayers, from 14 nm for films prepared from 10 mol % polymer to 25 nm for 100 mol % polymer vesicles. Using a viscoelastic model (which is more appropriate for less rigid films) to fit the frequency and dissipation data from the different sensor crystal harmonics, we determined the average thicknesses of hybrid films prepared from hybrid vesicles at pH = 2 were similar: approx. 15 nm for films prepared from 10 mol% polymer vesicles and up to 31 nm for 100 mol %. At pH = 5, the average thicknesses were similar for films prepared from 10, 25, and 50 mol % polymer vesicles as at pH = 2. However, thicknesses of films prepared from hybrid vesicles with higher fractions of polymer, 75 and 100 mol %, were much greater, and suggested adsorbed vesicles rather than bilayer films. 

For hybrid vesicles with higher polymer fractions, the thicknesses estimated from QCM-D data were greater than the maximum thickness of a membrane comprised of fully extended polymer chains. The 50 mol % polymer vesicles were only slightly thicker, 18–21 nm. The 75 and 100 mol % polymer vesicles yielded films with effective thicknesses double the expected thickness at pH = 2, and significantly higher at pH = 5, where the maximum hybrid polymer vesicle interactions with glass were observed. This increase in apparent thicknesses coincided with a substantial increase in dissipation between pH = 2 and pH = 5: from 10 × 10^−6^ to 14 × 10^−6^ for 75 mol % polymer vesicles and from 5 × 10^−6^ to >16 × 10^−6^ for 100 mol % polymer vesicles (for the 3rd overtone, or n = 3). The jump for pure polymer vesicles is especially striking, and film morphologies of supported polymer bilayers at low pH and adsorbed polymer vesicles at pH = 5. 

The thicknesses of 10 and 25 mol % polymer hybrid bilayers estimated from QCM-D data are greater than ~6.5 nm membrane thicknesses measured previously by cryogenic transmission electron microscopy (cryo-TEM) [31]. This discrepancy between measurements using QCM-D and cryo-TEM is not surprising because soft materials, particularly polymers that couple strongly to water like PEO, have similar contrast to the surrounding fluid and do not show up well in cryo-TEM. Nevertheless, the presence of the polymer, even in small amounts, could result in membranes that approach the thickness of the nearly fully extended EO_22_Bd_33_ on the order of 13–14 nm [44,45]. Fully extended polymer chains would allow for the trapping of water between the membrane and the glass substrate on the bottom of a supported bilayer membrane, and also exhibit significant coupling of the outer PEO chains with the surrounding water on the top of the supported bilayer membrane. The result would be an apparent thickness as determined by QCM-D that is greater than what would be observed by cryo-EM.

## 4. Conclusions

In this study, we varied vesicle composition and solution pH in order to generate a broader picture of spontaneous hybrid lipid/PEO-PBd vesicle interactions with glass supports. Using quartz crystal microbalance with dissipation (QCM-D), we followed the interaction of hybrid lipid-polymer vesicles with borosilicate glass as a function of pH. We found pH-dependent adsorption/fusion of hybrid vesicles that accounts for some of the contradictory results observed in previous studies. Specifically, it is now clear that pH has a significant effect on the adsorption/fusion of vesicles that contain polyethylene oxide blocks: in some cases, irreversible adsorption is observed, and in other cases there is no observable adsorption. Our results show that the formation of hybrid DOPC/EO_22_Bd_33_ bilayers can be tuned with pH, and that the adsorption/fusion sensitivity to pH is particularly obvious near pH = 7. The QCM-D results for films prepared from hybrid vesicles at low pH (pH = 2) are consistent with supported bilayers rather than adsorbed vesicles, though we cannot rule out the possibility of a heterogeneous film of both bilayers and adsorbed vesicles. Our results are limited to a specific lipid/polymer combination, and one of many possible buffers (Tris), but we expect that they will be relevant for a wide range of buffer conditions and lipid/polymer combinations that involve polyethylene oxide chains. We anticipate that our understanding of pH and other factors involved in the formation of supported bilayers will greatly expand our ability to create biomimetic bilayers as model surfaces and platforms for more robust biosensor devices. 

## Figures and Tables

**Table 1:** Characterization of hybrid lipid/polymer vesicles.

Sample	DOPC (mol %)	EO_22_Bd_33_ (mol %)	Diameter (nm) ^a^	PDI ^a^	ζ (mV) ^b^
0%	100	0	70.9 ± 0.2	0.11 ± 0.01	−3.4 ± 0.2
10%	90	10	74 ± 1	0.21 ± 0.01	−2.1 ± 0.7
25%	75	25	78 ± 2	0.23 ± 0.02	−3.2 ± 0.6
50%	50	50	104 ± 3	0.19 ± 0.01	−1.9 ± 0.4
75%	25	75	116 ± 2	0.18 ± 0.02	−2.5 ± 0.5
100%	0	100	131 ± 1	0.18 ± 0.01	−2.3 ± 0.4

^a^ Z-Average diameter and PDI from cumulants analysis ± standard deviation of at least 3 measurements, taken from vesicles after 11 passes through a 50 nm polycarbonate membrane. ^b^ ζ measured in Tris buffer solution at pH = 7.0.

**Table 2:** Estimated thickness of hybrid films.

Sample ^a^	Sauerbrey Thickness at pH = 2 (nm) ^b^	Broadfit Thickness at pH = 2 (nm) ^c^	Broadfit Thickness at pH = 5 (nm) ^c^
0%	5.3	^d^	6
10%	14	15	13
25%	14	15	13
50%	18	20	21
75%	24	26	56
100%	25	31	50

^a^ The samples are films prepared from hybrid EO_22_Bd_33_/DOPC vesicles where the name of the Sample is the mol % of polymer in DOPC. ^b^ Estimated from the 3rd overtone using the Sauerbrey equation assuming a rigid film and a film density of 1 g/cm^2^. ^c^ Estimated from frequency and dissipation data using a frequency dependent viscoelastic model (Broadfit, Dfind software, Nanoscience Instruments). ^d^ Broadfit model did not converge.

## Supplementary Materials

The following are available online at https://www.mdpi.com/2073-4360/12/4/745/s1, Figure S1: Zeta potential as a function of pH for hybrid DOPC vesicles with different mole fractions of EO22Bd33 from Figure 1 in the main manuscript plotted individually with error bars, which represent the standard error for a set of at least 3 measurements; Figure S2: Zeta potential as a function of pH for glass microspheres from Figure 1 in the main manuscript plotted with error bars, which represent the standard error for a set of at least 3 measurements.
