# Supplementary material for: Hybrid Lipid-Polymer Bilayers: pH-Mediated Interactions between Hybrid Vesicles and Glass
Source: Polymers (Basel). 2020 Mar 28;12(4):745. doi: 10.3390/polym12040745 (PMC7240632; doi:10.3390/polym12040745)
Supplement: Supplementary file 1 [file polymers-12-00745-s001.pdf]

Supporting Information for:

Hybrid Lipid-Polymer Bilayers: pH-Mediated Interactions Between Hybrid Vesicles and Glass

Keith L. Willes[1], *Jasmyn R. Genchev*[2] *and Walter F. Paxton*[1,*]

[1] Department of Chemistry and Biochemistry, Brigham Young University, Provo, UT 84602

[2] Northern Arizona University, Flagstaff, AZ 86011

[*]Corresponding author

Walter F. Paxton
Department of Chemistry and Biochemistry
BNSN C-100
Brigham Young University
Provo, UT 84602 USA
Tel: +1 (801) 422 4917
Fax: +1 (801) 422 0153
E-Mail: paxton@chem.byu.edu

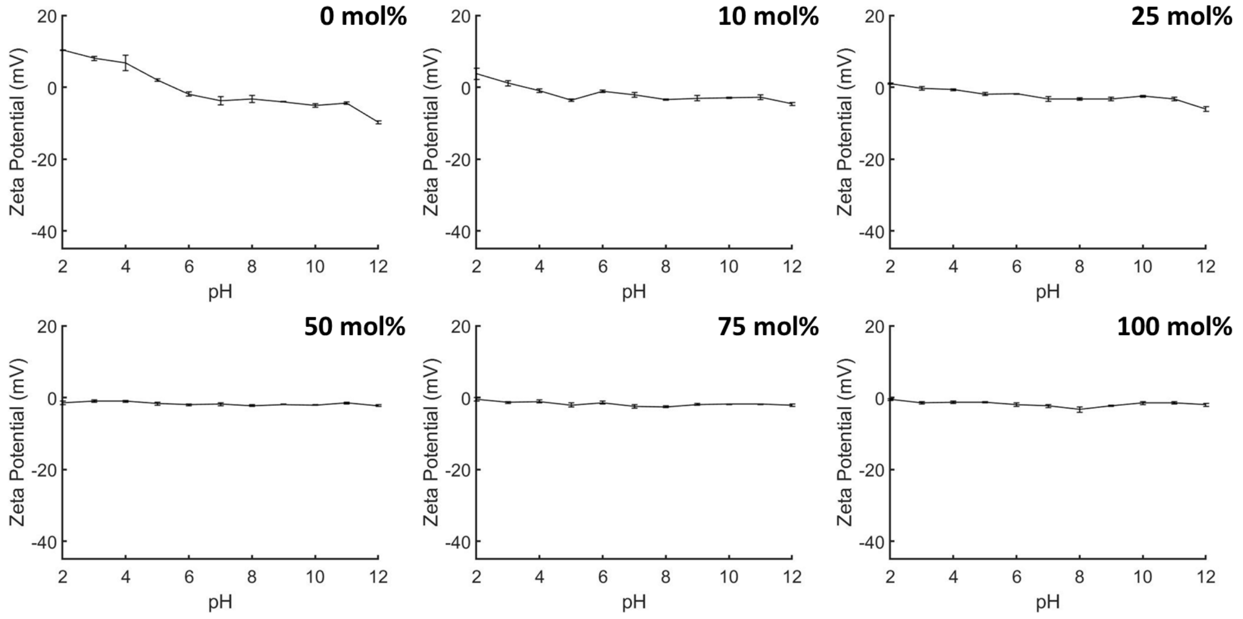

Figure S1. Zeta potential as a function of pH for hybrid DOPC vesicles with different mole fractions of $EO_{22}Bd_{33}$ from Figure 1 in the main manuscript plotted individually with error bars, which represent the standard error for a set of at least 3 measurements.

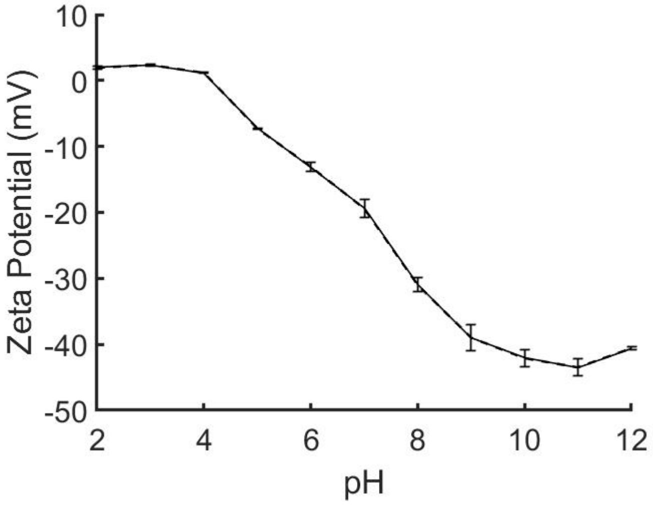

Figure S2. Zeta potential as a function of pH for glass microspheres from Figure 1 in the main manuscript plotted with error bars, which represent the standard error for a set of at least 3 measurements.
